# Erythroderma, Alopecia, Anhidrosis, and Vitiligo as Complications of a Red Ink Tattoo—A Case Report

**DOI:** 10.3390/clinpract15120224

**Published:** 2025-11-28

**Authors:** Mateusz K. Mateuszczyk, Magdalena Łyko, Andrzej Bieniek, Joanna Maj, Alina Jankowska-Konsur

**Affiliations:** University Centre of General Dermatology and Oncodermatology, Wroclaw Medical University, 50-556 Wroclaw, Poland

**Keywords:** tattoo complication, tattoo, erythroderma, alopecia, anhidrosis, vitiligo, case report

## Abstract

**Background:** Adverse reactions to tattoo pigments are increasingly recognized, yet severe systemic complications remain rare and poorly characterized. Red tattoo ink, in particular, is associated with delayed hypersensitivity reactions, but widespread manifestations affecting multiple organ systems have not been documented. This case report aims to describe an unusual constellation of erythroderma, alopecia universalis, anhidrosis, and vitiligo triggered by red tattoo ink and to highlight the diagnostic and therapeutic challenges associated with such reactions. **Case presentation:** This case report describes a 36-year-old Caucasian male who developed erythroderma, alopecia, anhidrosis, and vitiligo as complications of a red ink tattoo, marking a rare and previously unreported case of such extensive reactions. Four months after getting the tattoo, the patient began to develop itchy eczematous changes, which progressed to erythroderma over several months, along with alopecia universalis and anhidrosis. **Results:** After months of ineffective treatment with glucocorticosteroids, cyclosporine, methotrexate, and acitretin, patch tests confirmed hypersensitivity to possible components of the red tattoo ink, prompting surgical removal of the inflamed tattoo fragments. Unfortunately, aside from resolving the erythroderma, this did not improve the patient’s clinical condition, and he developed vitiligo. Only after the complete removal of the red tattoo ink from the same series that caused the sensitization and the use of immunosuppressive and immunomodulatory drugs, including JAK inhibitors, was hair growth restored and the progression of vitiligo halted, but with no effect on anhidrosis. **Conclusions:** This case highlights the challenges in managing systemic reactions to tattoo ink and the importance of thorough evaluation and treatment strategies.

## 1. Introduction

Tattoos have been an integral part of human culture for centuries, serving as symbols of identity, expression, and artistry. However, alongside their popularity, tattoos carry the risk of various complications, ranging from mild skin irritations to more severe systemic reactions. This case report presents a unique and extensive manifestation of complications arising from a red ink tattoo in a 36-year-old Caucasian male, marking a rare case of such reactions. In light of this case, there is a growing recognition of the need for regulations governing tattoo ink composition, as well as education aimed at both tattoo artists and the public regarding the safety of tattooing. By addressing these aspects, we aim to reduce the incidence of complications associated with tattoos and improve the management of adverse reactions when they occur.

## 2. Case Presentation

**Figure 1:**
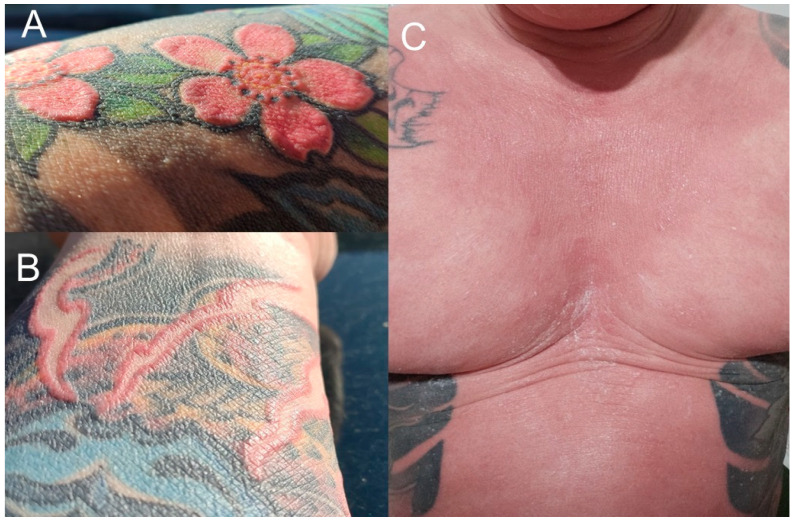
Initial stage of the disease: (**A**,**B**) skin changes within the red ink of the tattoo on the right forearm; (**C**) erythroderma and alopecia universalis.

In late July and August 2020, a 36-year-old Caucasian male had a tattoo done on his right forearm, using, among others, red pigment. About four months later, he experienced generalized itching with intensity 8–10/10, excessive skin exfoliation, and a rash consisting of nodules on a red background, initially affecting the forearms and chest. With the disease progressing, the eruptions spread to other parts of the body and the nodules began to merge into plaques. By January 2021, erythroderma had developed (Figure 1C). Periodically, palmoplantar keratoderma and dystrophic nail changes (hyperkeratosis and yellowing) were noticed. In February 2021, alopecia universalis and hypohidrosis occurred. Complete loss of sweating (anhidrosis) occurred three months later. As a result, from January to August 2021, the man was hospitalized multiple times in Dermatology, Hematology, and Internal Medicine Departments (the latter due to SARS-CoV-2 infection). Numerous histological examinations of skin biopsies with immunophenotyping were performed to exclude mycosis fungoides, pityriasis rubra pilaris, and erythrodermic psoriasis. The overall picture was consistent with eczema. Furthermore, an enlarged lymph node in the groin was biopsied, and its microscopic appearance corresponded to the diagnosis of dermatopathic lymphadenopathy. Based on the overall clinical picture, contact eczema with a tendency to erythroderma was the most likely diagnosis. The patient was chronically treated with oral glucocorticosteroids with a transient good clinical response, but during dose reduction, the skin lesions worsened to erythroderma. Additionally, oral cyclosporine, oral acitretin, and subcutaneous methotrexate were used in treatment, achieving periodic clinical improvement. From August to November 2021, the patient remained under the care of the Dermatology and Allergology Outpatient Clinic. During that time, immediately after discontinuation of systemic treatment, eruption-like changes were observed within the red pigment of the tattoo on the right forearm (Figure 1A,B). Patch tests confirmed type IV hypersensitivity to potassium dichromate, a mixture of kain, formaldehyde, and a fragrance mix. An allergologist recommended surgical removal of the tattoo. Therefore, from 10 November 2021, to 23 June 2022, 7 surgical procedures were performed, at the Plastic Surgery Clinic of the University Hospital in Łódź, to remove tattoo fragments containing pigment affected by the disease process. With each excision of tattoo fragments, the skin changes that caused numerous hospitalizations improved, but anhidrosis and alopecia persisted. In early July 2022, the first vitiligo patches appeared, which started to progress, partially covering areas of the entire body. Therefore, in August 2022, the patient first came to our Department and was hospitalized a total of 8 times.

**Figure 2:**
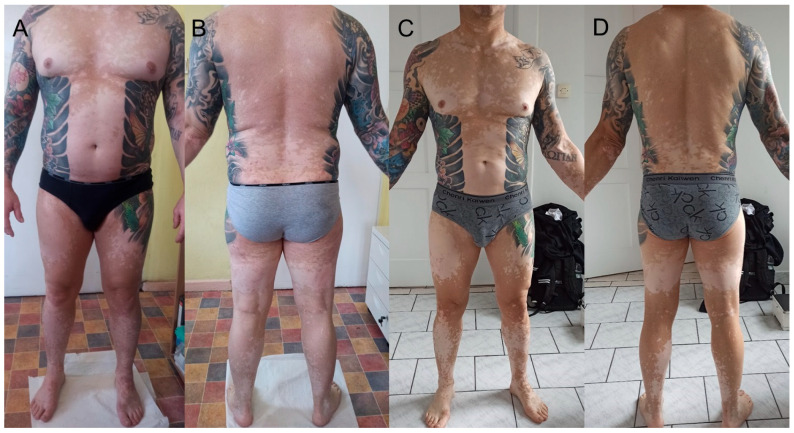
Body appearance over the course of the disease: (**A**,**B**) in 2022; (**C**,**D**) in 2024.

During the initial examination, alopecia universalis, anhidrosis, and vitiligo covering approximately 30% of the body surface were observed, as well as numerous scars on the right forearm—the result of tattoo fragment removal (Figure 2A,B). Moreover, the patient has a long history of Hashimoto’s thyroiditis and has been taking oral levothyroxine. The man reported significantly reduced exercise tolerance and was unable to perform any work due to the risk of heat stroke. The interview regarding the causes of central and neuropathic anhidrosis, peripheral causes arising from abnormalities in the sweat glands, and drugs causing it was negative. Detailed diagnostics were performed, and laboratory test results showed elevated levels of total IgE, CEA, anti-TPO, and anti-TG, with normal levels of TSH, and a positive result in ANA1 testing, where antibody levels were 1:320 for speckled, nucleolar, and cytoplasmic types, with simultaneous negative result in ANA3. Imaging studies, including chest X-ray, ultrasound of peripheral lymph nodes, salivary glands, head MRI with contrast, and colonoscopy, did not reveal significant deviations. Consultations with endocrinologists, neurologists, and ophthalmologists were also conducted, which did not reveal any abnormalities. During the last consultation, the Schirmer test was performed: 6 mm for the right eye and 30 mm for the left eye, indicating a negative result for Sjögren’s syndrome. The Minor test was negative. Unfortunately, we were unable to analyze the chemical composition of the tattoo ink because the man did not obtain a sample or the packaging from the tattoo artist.

In the differential diagnosis, we considered anhidrosis, total alopecia, and vitiligo as complications of erythroderma in the course of generalized contact eczema and acquired idiopathic generalized anhidrosis (AIGA).

**Figure 3:**
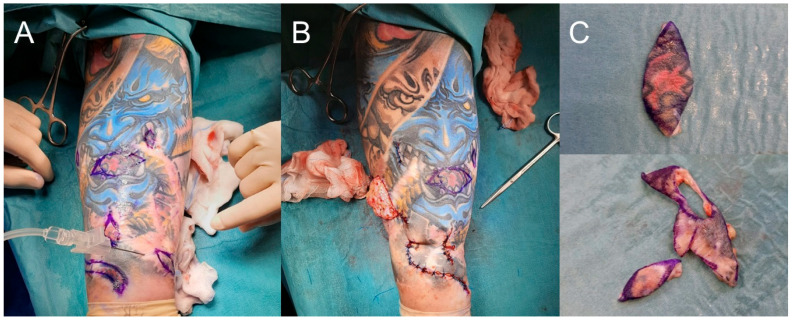
Surgical removal of the remaining fragments of the tattoo with red pigment: (**A**) anesthesia; (**B**) partial excision; (**C**) excised tattoo fragments.

Initially, cyclosporine was administered at a dose of 400 mg/day orally (5.8 mg/kg ideal body weight), with good tolerance, and the results of follow-up tests and blood pressure measurements remained normal throughout the therapy. Just a few weeks into treatment, the first hair regrowth was achieved, and after 6 months, it was almost complete. The progression of vitiligo was halted; however, no repigmentation process was observed. The Minor test remained negative. Due to the lack of sweating return in October and November 2022, a total of 2 pulses (3 infusions of 500 mg) of methylprednisolone intravenously were administered. At that time, the cyclosporine dose was reduced to 300 mg/day. Unfortunately, the Minor test remained negative, and there was no repigmentation of vitiligo patches. A skin biopsy of the foot sole was performed due to lack of response to aggressive treatment, and histology confirmed several normally structured sweat glands. It is worth mentioning that the CEA level decreased over time from 12.83 ng/mL in August 2022 to 4.12 ng/mL in February 2023. In January 2023, the patient reported the appearance of new nodular lesions within the area of the previously untouched red pigment of the tattoo on the right forearm. A surgical procedure was performed to remove this fragment, as well as the remaining visually unchanged part with red pigment (Figure 3A–C). The histological examination supported the diagnosis of eczema. Despite the above, there was no restoration of sweating or normal pigmentation. Therefore, cyclosporine was discontinued and switched to baricitinib at a dose of 4 mg/day orally, which is still being continued to this day. As a result of the therapy, there was accelerated hair regrowth, and inhibition of vitiligo progression, but with no effect on anhidrosis (Figure 2C,D). The Minor test remained negative.

**Figure 4:**
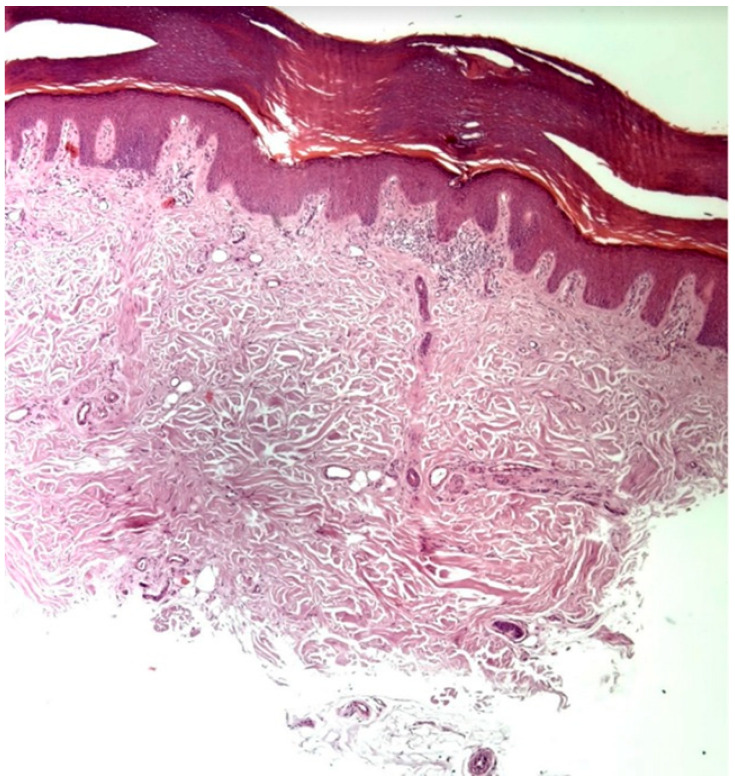
The histological image of the skin biopsy from the right hand shows destruction and subsequent fibrosis of the eccrine glands and their excretory ducts (40×).

Further skin biopsies from unaffected skin from the right hand and left armpit were conducted to assess the presence and structure of sweat glands. The histology report of the right hand sample revealed a markedly reduced number of eccrine glands, with those present displaying signs of atrophy and ductal fibrosis, attributed to fibrotic bands oriented perpendicular to the epidermis. The number of acrosyringia was also significantly diminished, with the remaining structures showing abnormal keratinization and dyskeratotic cells. This process appears complete. Scattered lymphocytes and mast cells were present around the few remaining eccrine glands, which exhibited signs of atrophy. The microscopic examination thus indicated destruction with subsequent fibrosis of the eccrine glands and their ducts (Figure 4). Similarly, in the sample from the left armpit, a loss of eccrine sweat glands was observed. Additionally, a sample of the fragment of the tattoo from the right forearm was taken, but from the area of the blue pigment, to assess whether there was a hypersensitivity reaction to components of the tattoo other than the red pigment. No histological features indicating a hypersensitivity reaction were described, and additionally, no acrosyringia were found. These findings suggest a very low likelihood of sweat function recovery, although complete irreversibility cannot be stated with absolute certainty. Our treatment focuses on therapy for vitiligo and alopecia.

The patient is using a water spray bottle to cool down his body. He has noticed that since he developed anhidrosis, his diuresis has increased, which coincided with an improvement in his body’s exercise performance.

## 3. Discussion

A tattoo is a form of body modification involving the introduction of a mixture of chemical substances into the dermis to change its color [1]. Although precise epidemiological data are lacking, estimates suggest that about one in four young people living in the USA or Europe has at least one tattoo [2]. Like any procedure, it carries the risk of complications. In one survey study of 3601 participants, 67.5% reported some skin problems associated with getting a tattoo [3]. Research shows that the number of adverse reactions depends, among other factors, on the color of the dye used, which in turn depends on the composition [4]. Most commonly they concern red ink [4]. It is worth noting that until 2022, there were no regulations regarding the composition of tattoo ink in the European Union (EU), and in many countries around the world, regulations still do not exist. Before legislative solutions were introduced, red ink commonly contained compounds such as mercury, cadmium, and arsenic [4]. These substances are commonly toxic and carcinogenic [4]. Since January 2022, the use of dangerous chemicals in tattoo inks has been restricted in the EU under the REACH regulation [5]. We believe this will contribute to reducing the number of reported adverse reactions. The patient, in the clinical case we are describing, got a tattoo in 2020, two years before these regulations were introduced. Therefore, the composition of the tattoo ink may have contained compounds that are currently prohibited. According to the REACH regulations, certain chromium compounds as well as formaldehyde may be used in tattoos [5]. The patient described had a hypersensitivity reaction in patch tests to the substances described above. However, chemical analysis of the specific red tattoo ink in our case could not be performed, as the original sample was unavailable, and the above findings cannot definitively confirm the presence of such substances in the ink itself. Therefore, despite the compliance of the tattoo with European standards, the patient could still develop adverse reactions. This highlights the importance of taking a detailed medical history from the patient before performing a tattoo, including inquiries about allergic diseases. In cases with a positive history of such conditions, we believe it should be standard practice to perform patch tests with the specific tattoo ink to be used. Patients should also be informed that patch tests in these cases sometimes yield negative results despite the possibility of developing hypersensitivity reactions [6,7]. This is most likely due to the need for haptenization, or because the reaction occurs after the substance is introduced into the dermis rather than through contact with the epidermis [6]. Patients must give informed consent. Perhaps there is a need for regulation that would govern the issues of education, training, and granting qualifications allowing professional practice as a tattoo artist. Currently, such regulations don’t exist in Poland. Furthermore, patient education may partially reduce the scale of the problem by decreasing the number of tattoos performed, especially in individuals with contraindications. There are many types of adverse reactions associated with getting a tattoo, particularly with red ink. These reactions can be categorized into 5 groups: inflammatory, infectious, neoplastic, aesthetic, and miscellaneous [4]. Although they differ from each other, their common starting point is the tattooing mechanism, which is worth briefly discussing [4]. Using a needle, the pigment is placed into the dermis [4]. Damage to the basal membrane and the epidermal and dermal cells activates an acute inflammatory reaction, clinically presenting as transient erythema [4]. During the healing process, microorganisms can penetrate through the damaged skin structure, leading to infection [4]. After the acute inflammatory reaction subsides, there is activation of a reaction to the foreign material [4]. The pigment particles are insoluble and resistant to degradation, so they remain in the dermis [4]. Smaller tattoo particles can penetrate deeper into the dermis, where they are captured by Langerhans cells and transported to the lymph nodes, causing dermatopathic lymphadenopathy (this coincides with the biopsy of our patient’s lymph node) [4]. In some individuals, this process may be dysregulated or overly intense, leading to adverse reactions [4]. Research indicates that hypersensitivity reactions to red tattoo pigment are most likely a combination of delayed hypersensitivity type IVa and type IVc allergic reactions [8]. Biopsy of skin lesions in such cases is helpful, especially in less obvious cases like our patient’s, where histologists typically describe predominantly histiocytic infiltrates in the dermis and epidermal interface dermatitis [8]. Chronic allergic reactions to tattoos can occur months or even years after getting the tattoo [9]. 

Typically, the skin changes are confined to the tattooed area, but very rarely, as in our patient, they can become generalized [10]. Apart from our patient, we found only one case of generalized skin changes associated with red tattoo ink in the literature [6]. A previous publication by Szulia et al. [10] in 2022 reported the early phase of this same patient’s course, focusing on the acute systemic allergic reaction and partial surgical management. The present report extends that description by providing a detailed long-term follow-up and documenting the development of alopecia universalis, persistent generalized anhidrosis, progressive vitiligo, comprehensive eccrine gland histopathology, and the real-world outcomes of systemic immunosuppressive and immunomodulatory therapy, including JAK inhibition, and outcomes after excision of all red pigment. Therefore, the novelty of this paper lies not in the initial reaction itself, but in the unique constellation of long-term complications and the full clinical trajectory observed over four years.

Scientific data indicates that ¼ of patients diagnosed with an autoimmune disease will develop another [11]. Case reports have been described in the literature of hypersensitivity reactions to red tattoo ink in patients with conditions such as atopic dermatitis, asthma, and celiac disease [12]. Additionally, cases have been reported of the initial onset of symptoms of autoimmune diseases following tattooing, such as myopathic dermatomyositis, discoid lupus erythematosus, and sarcoidosis [12]. To date, there has been a single case reported in the global medical literature describing coincidence of a lichenoid red tattoo reaction and alopecia areata [13]. Affected tattoo areas were treated with intradermal triamcinolone [13]. Even though alopecia areata patch was not directly treated, restoration of hair was observed [13]. This suggests the existence of a common pathomechanism for these diseases, which would explain why these individuals are more susceptible to developing various hypersensitivity-related conditions. Therefore, patients with some autoimmune diseases, like our patient with Hashimoto’s thyroiditis, should be particularly cautious when deciding to get a tattoo. Given the patient’s history of Hashimoto’s thyroiditis, the red tattoo ink may have acted as an external trigger in an individual with autoimmune predisposition rather than as a direct cause of alopecia, vitiligo, and anhidrosis. Although a temporal association between tattooing and the onset of erythroderma was observed, other potential triggers cannot be completely excluded, and the coexistence of these events may be coincidental. Therefore, the causal relationship between the tattoo and the systemic reaction should be interpreted with caution. To the best of our knowledge, a case of erythroderma, total alopecia, generalized anhidrosis, and vitiligo as complications of red tattoo ink has not been described before.

In cases of severe or systemic hypersensitivity reactions, surgical removal of all tattooed areas containing the suspected allergenic red pigment may be considered. In our experience with this patient, full-thickness excision with direct closure or grafting appeared to be the most reliable way to eliminate the suspected trigger. However, this should be understood as expert opinion based on a single, exceptional case rather than a general recommendation. As in the case of our patient, a few months after removing the inflamed part of the tattoo, inflammation began to develop in the remaining part. This might have been the reason for the ineffectiveness of alopecia areata and anhidrosis treatment and the development of vitiligo. Considering that delayed hypersensitivity reactions to tattoos can develop at different times, even in the same patient, the above-described approach is warranted [9]. Other forms of treatment, such as topical or systemic corticosteroids, are usually ineffective, and laser therapy is contraindicated due to the possibility of photochemical induction of new allergen production during tattoo removal with this method [10]. Dermabrasion and dermatome shaving are good methods if we know that the allergenic ink is located superficially, which is difficult to predict, since deeper removal may cause significant scarring [10]. Therefore, it has certain limitations.

The association between red ink hypersensitivity and anhidrosis remains unclear. A possible mechanism involves immunologic cross-reactivity between tattoo pigment components and eccrine gland antigens, leading to lymphocytic infiltration and cytokine-mediated glandular damage. Histopathological findings in our patient, showing perieccrine inflammation and partial glandular atrophy, support this hypothesis. Previous studies have demonstrated impaired sweating in tattooed skin, suggesting functional alteration of eccrine structures [14], while autoimmune mechanisms involving T-cell infiltration around sweat glands have been described in acquired idiopathic generalized anhidrosis [15]. These data support the concept that tattoo-related immune activation may extend beyond the local site, contributing to systemic dysfunction.

Functional assessment with the Minor test was persistently negative throughout the disease course, indicating a marked reduction of sweating; however, this method evaluates only the macroscopic presence of sweat and may not detect minimal or regionally preserved eccrine activity. Anhidrosis can occasionally present with patchy or segmental involvement [16], and therefore a negative Minor test should be interpreted as strong but not absolute evidence of global sweat loss. In this context, the histopathological findings of markedly reduced eccrine glands and ductal fibrosis are highly suggestive of advanced and likely long-standing tissue injury, yet they do not entirely exclude the possibility of limited residual gland function.

As there were promising data regarding the use of baricitinib in vitiligo and alopecia areata, we decided to initiate treatment with this JAK inhibitor. Recently, a retrospective controlled study on baricitinib and nb-UVB in vitiligo, as well as a 52-week multicenter retrospective real-world study on baricitinib use in alopecia areata, were published [17,18]. Both studies confirmed the effectiveness of this treatment option. Since the patient started taking baricitinib, hair regrowth was observed and vitiligo has stabilized; however, no repigmentation of existing lesions has been observed.

Although the patient’s malignancy workup was negative, the observed elevation of CEA may reflect chronic systemic inflammation, as increased CEA levels have been reported in various inflammatory and autoimmune dermatoses. Additionally, we do not know the underlying cause of the correlation between decreasing CEA levels over time during treatment of our patient, but similar observations have been described in cases of AIGA, suggesting that CEA reduction may parallel the resolution of inflammatory activity.

In summary, due to the small number of published cases, it is not possible to develop a management scheme other than the one described above concerning surgical treatment.

## 4. Conclusions

Managing systemic reactions to red tattoo ink is complex and challenging. While surgical removal of affected tattoo fragments is important, complete removal of sensitizing pigment, in our opinion, should be considered to prevent recurrence and the development of complications. The association between allergic and autoimmune diseases and adverse reactions to tattoo ink suggests a need for increased awareness and caution, particularly in susceptible individuals. In patients with a history of autoimmune, dermatological or allergic diseases, a dermatological consultation is recommended prior to undergoing tattooing. Perhaps regulations regarding licensing for tattoo artists and education on the safety of tattooing will contribute to reducing the number of complications associated with them.

## Data Availability

The original contributions presented in this study are included in the article. Further inquiries can be directed to the corresponding author.
